# Challenges for co-morbid chronic illness care and policy in Australia: a qualitative study

**DOI:** 10.1186/1743-8462-6-22

**Published:** 2009-09-08

**Authors:** Tanisha Jowsey, Yun-Hee Jeon, Paul Dugdale, Nicholas J Glasgow, Marjan Kljakovic, Tim Usherwood

**Affiliations:** 1 The Australian Primary Health Care Research Institute, Building 62, Mills Road, The Australian National University, Canberra, Australia; 2 Centre for Health Stewardship, The Australian National University and Chronic Disease Management Unit, ACT Health, Canberra, Australia; 3 Medical School, The Australian National University, Canberra, Australia; 4 School of General Practice, Rural, & Indigenous Health, Medical School, The Australian National University, Canberra, Australia; 5 Discipline of General Practice, Sydney Medical School - Western, The University of Sydney, Sydney, Australia

## Abstract

**Background:**

In response to the escalating burden of chronic illness in Australia, recent health policies have emphasised the promotion of patient self-management and better preventive care. A notable omission from these policies is the acknowledgment that patients with chronic illness tend to have co-morbid conditions. Our objectives were: to identify the common challenges co-morbidity poses to patients and carers in their experiences of self-management; to detail the views and perceptions of health professionals about these challenges; and to discuss policy options to improve health care for people with co-morbid chronic illness. The method included semi-structured interviews and focus groups with 129 purposively sampled participants. Participants were people with Type 2 diabetes, chronic obstructive pulmonary disease and/or chronic heart failure as well as carers and health care professionals. Content analysis of the interview data was conducted using NVivo7 software.

**Results:**

Patients and their carers found co-morbidity influenced their capacity to manage chronic illness in three ways. First, co-morbidity created barriers to patients acting on risk factors; second, it complicated the process of recognising the early symptoms of deterioration of each condition, and third, it complicated their capacity to manage medication.

**Conclusion:**

Findings highlight challenges that patients with multiple chronic conditions face in relation to preventive care and self-management. Future clinical policy initiatives need to move away from single illness orientation toward strategies that meet the needs of people with co-morbid conditions and strengthen their capacity to self-manage. These patients will benefit directly from specialised education and services that cater to the needs of people with clusters of co-morbidities.

## Background

In response to the escalating burden of chronic illness in Australia, the Council of Australian Governments instigated the Better Health for All Initiative [1] in line with the 2005 National Chronic Disease Strategy [2]. Central to these changes is the promotion of patient self-management and better preventive care through increased patient support to act on risk factors [3,4]. State and territory governments have developed policies consistent with this. At federal, state and territory government levels, policy initiatives are primarily single-illness oriented and this is reflected in many aspects of organisation planning and delivery of health services [5]. A notable omission from these policies is the acknowledgment that patients with chronic illness tend to have co-morbid conditions, the prevalence of which increases with age [6-8]. The relationship between chronic heart failure (CHF) and co-morbid depression is well-established [9,10]. However, a preliminary review of the literature indicates few studies address the impact of other co-morbid conditions on chronic disease management.

The Serious and Continuing Illnesses Policy and Practice Study (SCIPPS) is a five-year National Health and Medical Research Council-funded research program that focuses on better policy for improving patient experience in managing chronic illness. Three conditions--Type 2 diabetes mellitus (DM), chronic obstructive pulmonary disease (COPD), and chronic heart failure CHF--were studied as they have a high prevalence in Australia, and for each one clinical prevention is known to be effective. A qualitative study that forms the basis for this paper was undertaken involving 52 patients, 14 carers and 63 health care professionals (HCP). Eighty-seven per cent of patients indicated they had more than one chronic illness. Without being prompted by the interviewer, 55 of the 66 patients and carers raised co-morbidity as a complicating factor in their experience of chronic illness (and in response to prompting, a further two patients discussed co-morbidity as a complicating factor). Common patterns emerged from interrogation of the co-morbidity data and these patterns are reported in this paper, which has the following aims:

• To describe the common challenges co-morbidity poses to patients and family carers in their experiences of managing chronic illness

• To report HCP perspectives on these challenges

• To discuss the policy challenges these findings pose.

## Methods

The study used a generic qualitative approach [11] to explore the experiences and perspectives of patients with DM, COPD and/or CHF, family carers and health care professionals. Data collection and analysis were carried out by a group of seven research workers with multidisciplinary backgrounds in health and social sciences, all of whom trained as a group in workshops and followed a data collection manual to ensure consistency in data collection and analysis.

The definition of co-morbidity used in this study was "the coexistence of chronic conditions within the context of an index condition" [12]. The target population of this study were people affected by DM, COPD and/or CHF; therefore, in this paper, 'index condition' refers to those conditions. When patients had two or three index conditions, the conditions were counted as co-morbid and data were analysed in terms of problems that occurred as a result of the patient having two or more conditions.

### Sample

We used purposive sampling in order to obtain a range of patients and carers with varied demographics including age, ethnicity and severity of the illness. Patients and carers were recruited through referrals from general practices, local hospitals, community health services, specialist clinics, health care consumer organisations, as well as Aboriginal health services located in the Australian Capital Territory (ACT) and western suburbs of Sydney in Australia. Eligible participants included patients aged between 45 and 85 with one or more of the three conditions of interest (DM, COPD and CHF), who at the time of interview were living in either the ACT or western Sydney and did not have diagnosed cognitive impairment and family carers.

Health care professionals who had specific experience in the management of the index conditions were recruited through Divisions of General Practice and Area Health Services to include hospital specialists, general practitioners, nurses and allied health professionals. HCPs were included to provide contextual insight on the health system.

### Procedure

Study approval was obtained from the relevant institutional human research ethics committees and all participants provided informed consent prior to their participation. Data collection occurred between March 2007 and January 2008. Semi-structured in-depth interviews were conducted with patients and with carers; each interview running between 45 and 90 minutes. Patients and carers then completed a 10-minute demographic survey, which contained information about patient health conditions and health care encounters. One question included in the survey was, 'Apart from the CHF, COPD and/or DM do you have any other health conditions? If yes, please list all conditions (chronic and acute) and describe how long you have had them.' Interviewers clarified survey questions as required and provided practical assistance in completing the surveys.

All health care professionals participated in one-hour focus groups, with the exception of two HCPs who were interviewed separately. HCPs completed a two-minute survey about the extent and duration of their work in chronic illness. Although many of the HCPs who recruited patients and carers to the study also participated in the focus groups, the individual patient/carer data were not designed to be linked to HCP data. The research team judged sufficient data had been gathered when interviews and focus groups were no longer providing new insights or ideas deemed central to the experience of patients and carers, indicating data saturation [13].

### Interview questions

The patient and carer interviews began with a question asking the participant what it was like to live (or care for someone) with chronic illness. Other questions covered (but were not limited to) the most challenging aspects of their chronic illness, experiences with health services and health care providers and support they wanted and/or experienced in managing their chronic illness.

In their responses, many patients and their family carers raised challenges they faced in the management of chronic illness that was either caused, or made worse, by co-morbidity. Based on these findings co-morbidity was further explored with HCPs. Questions to HCPs concerning co-morbidity included: 'what are the main problems that people with multiple conditions face and what are the main reasons for the problems?'

### Analysis

All interviews and focus groups were electronically recorded and transcribed verbatim. The data were analysed using qualitative content analysis [13], assisted by a computerised qualitative data analysis program, QSR NVivo7 [14]. A coding scheme was created during the data collection phase and used to facilitate consistent data analysis by seven researchers across the two research sites. The coding scheme was refined by the collective researchers periodically throughout the data analysis and researchers regularly engaged in checking each other's interpretation accuracy of the data against the coding scheme. Strategies to ensure rigour were developed and adopted based on the work of Lincoln and Guba (1985) [15]. This included: extensive research worker training and practice in interview skills and data analysis; a pilot to assure adequacy of data collection and recruitment; development and meticulous implementation of data collection and analysis protocols; maintenance of inter-coder reliability according to established analysis protocol; and examination of qualitative data against relevant participant survey data. Descriptive analysis (frequencies, means, modes and medians) of the survey data was undertaken using SPSS version 15 [16]. In analysis of the co-morbidity data the first and second authors identified problems that participants experienced as a result of having two or more chronic health conditions.

## Results

### Sample characteristics

A total of 52 patients (male n = 28) and 14 carers (male n = 1) were recruited. Of these, 27 patients had DM, 17 patients had COPD and 20 patients had CHF. The total number combines to more than 52 because 10 patients had two of these conditions and one patient had all three conditions. This study succeeded in recruiting patients and carers from diverse cultural and ethnic backgrounds (n = 23), including seven Indigenous patients. Most patients and carers were older than 65 years (n = 42), experienced economic hardship (n = 42), had a decade-long history of chronic illness (mean = 16.5 years) and had monthly or more frequent contact with general practitioners (GPs).

Forty-five patients had co-morbid chronic illness. The common co-morbidities included the index conditions of arthritis, osteoporosis, asthma, and back pain. Depression and pneumonia were two conditions commonly discussed by patients; most of whom at the time of the interview did not have the condition but were mindful that they were prone to recurrences.

Of the 63 health care provider (HCP) participants, most were female (n = 44) and working full time (n = 55). The majority of HCP participants were registered nurses (n = 23) and physicians (n = 21, 15 GPs and six specialists). Other participating HCPs included physiotherapists, care coordinators, managers, occupational therapists, podiatrists, psychologists and social workers. The length of HCP work experience varied from less than one year to 33 years (median = 8 years). There were 12 HCP participants who listed their role in chronic disease management as "non-clinical work," meaning they were not involved in direct patient care, yet played a major role in the service provision for this population.

### Common challenges posed by co-morbidity

Co-morbidity increased the amount of time participants spent managing their health and increased patients' dependency on others. Patients with co-morbid conditions encountered problems with the coordination between services and with polypharmacy. Patients prioritised the management of one condition over another; consequently, some health issues could be neglected or compromised. The three most common challenges to patients and carers in managing chronic illness (either caused or made worse by co-morbidity), relate to acting on risk factors, recognising signs and symptoms of illness and managing medications. Table 1 indicates key issues that patients and carers discussed in connection with co-morbidity.

**Table 1:** Patient and carer perspectives on challenges associated with co-morbidity

**Challenges associated with co-morbidity**	**Co-morbidity (n = 57)**
Acting on risk factors	13

Recognising change in illness condition	31

Being on guard/alert to changes	11

Illness management mechanism (inc. medication)	35

Uncertainty	13

Learning	18

Planning (inc. medication & diet)	12

Adverse effects of medication	05

Remaining positive	13

Managing abnormal blood sugar levels	13

Complications in illness (caused by index condition)	13

Living with anxiety and fear	26

Obtaining quality care	21

Balancing life and illness	19

Physical or social activities	17

### Capacity to act on risk factors

Risk factors are variables known to increase a person's risk of illness or deterioration in health; the term 'risk factor' was used by HCPs but not by patients and carers. Common risk factors that patients and carers discussed included physical inactivity, poor diet, stress, smoking and excessive alcohol consumption. While patients and their carers indicated an awareness that changing their lifestyle would prevent further deterioration, co-morbidity frequently prevented them from acting on risk factors effectively--raising feelings of guilt, frustration, depression and anxiety.

Many patients found it difficult to maintain a healthy diet (a topic raised especially by patients who had both DM and CHF). Clinical depression was a common co-morbid condition that reduced patient motivation to follow a healthy diet or exercise. For some patients co-morbid conditions such as arthritis delayed completion of rehabilitation programs or caused them to withdraw from the program:

There were people older than me [in the cardiac rehabilitation program] and I couldn't keep up with them because of my ankle. That's how I went to the doctor and I showed them and I got arthritis. That was why I couldn't walk properly. ...I got an exercise bike. I look at it a lot, that bloody bike. Bit slack.

Man in his sixties with DM and CHF

Similarly, HCPs reported that co-morbid conditions, in particular arthritis and depression, could limit usefulness of rehabilitation programs for patients' index chronic conditions. Premature withdrawal of patients from rehabilitation programs due to co-morbid conditions was costly, as was extended participation in programs, which was perceived as creating further resource constraints in a system already under considerable strain:

So we can either treat the ones that we do very well and neglect all the ones who are trying to get in who also need it, or we ditch them before they've done their best work to try and get more in. You can't win either way. So that's again a lack of resources and availability.

HCP in the ACT

This perception of resource restraints explained why HCPs deterred people with multiple conditions from staying in rehabilitation programs.

### Capacity to recognise the signs and symptoms of illness

Co-morbidity made it difficult for patients to recognise signs and symptoms of the index condition, especially early warnings of an exacerbation. This concern was raised more frequently by patients with CHF than by patients with DM or COPD. A woman in her seventies with DM, asthma and CHF said: "It is very hard for me to say whether it is my heart that I am short of breath with or asthma." Learning about the features of both their index condition and co-morbid conditions took much longer than simply learning about the features of a single condition. A carer explained that with her mother's recent diagnosis of DM they remained unsure whether her mother's mini-strokes were symptoms of DM or indicating a separate condition:

We don't know actually [what caused the mini-strokes]. Well, she did have a couple of episodes, I don't know if they would have been the mini-strokes, if they would have been diabetes or what it would have been. But of course ...she was only diagnosed recently [with DM].

Daughter carer in her fifties of a woman with DM

Patients indicated they learnt how to recognise signs and symptoms of exacerbation by applying information gained through various sources (written sources, conversations with health professionals, friends and family) to their personal experience in a process of trial and error. A wife carer of a man with DM and Alzheimer's disease learnt over time to differentiate between the signs and symptoms associated with each illness:

Sometimes he'd get a little bit tired and in a second he'd fell and hit his head. He would slur his words and things like that but I knew he had the symptoms of no sugar. ...Over time, you just realise that this is what's going on.

Wife carer in her seventies of a man with DM

Patients said they wanted more information that addresses the links between co-morbid conditions to facilitate their self-management.

Health care professionals reiterated the difficulty for patients in recognising signs and symptoms of co-morbid conditions, noting that this is a particular problem for patients with limited health knowledge. HCPs further explained that even when patients did correctly identify new symptoms they did not always know how to respond and so ended up in hospital or suffered unnecessarily at home.

### Capacity to manage medications

Four out of five patients had co-morbid conditions. A total of 38 patients received treatment with medication for at least one of their conditions and 20 patients were prescribed seven or more medications, each with their own daily regimen. Patients found managing medication for their numerous conditions to be complicated, time-consuming, inconvenient and confusing. They raised concerns of insufficient knowledge about drug interactions and side-effects, and not being able to manage their medications. Others did not follow medication recommendations because they did not like taking pills. For example, a woman with DM and CHF did not take her prescribed medication for DM management because she did not want to increase the number of pills she was already taking (for management of CHF): "I'm on so many heart tablets and things like that, I didn't want to take any [more] medication, so I went for diet, and diet control." This is also an example of the patient prioritising treatment of one condition over another.

Many patients demonstrated limited knowledge and understanding of their medications and were unable to differentiate between them. A carer in her fifties of a woman with DM said: "I have to do the medicines these days. ...I kept noticing she didn't know what to call the tablets and stuff and now she's got over 20 tablets [daily]." Similarly, a farmer with DM and COPD said: "Well I'm not too sure what they're for but I know they're either for diabetes or for me heart, or cholesterol, or high blood pressure." While blister packs preloaded with medication were often perceived as helpful in managing medications, some people with the packs found they no longer knew what medication they were taking and could not distinguish between medications. Cognitive impairment or dementia further impaired their ability to manage, and in many cases their carers had taken over that task.

Patients discussed the complex process of finding suitable medications to manage their conditions, noting that often this required good communication with health care professionals, which in turn was dependent on patient awareness of signs and symptoms associated with their numerous conditions. HCPs raised other critical elements influencing medication compliance such as patient honesty or recall/forgetfulness about which medications they were actually taking. HCPs indicated financial constraints and the cost of filling scripts often caused patients with co-morbid conditions to skip medications they thought were less important than others. One HCP said:

They tend to pick and choose which... scripts they get filled, because they've got so many things going on at once... And the whole issue of medication management arises and it escalates their co-morbidity.

HCP in the ACT

Several health care professionals indicated that medication management and non-compliance were particular problems with patients with mental illness. They suggested that better access to mental health care providers could improve medication management for these patients. HCPs also suggested that lack of awareness by HCPs and patients concerning risks involved in using multiple medication brand names could lead to patients unknowingly taking doses higher than prescribed, resulting in ill health, and that this could go unnoticed. Patients, carers and HCPs suggested that the capacity to manage medication could be improved through increased education, patient engagement and good communication between patients and their HCPs.

## Discussion

Patients and their carers found co-morbidity limited their capacity to manage chronic illness by diminishing patient ability to reduce risks, recognise symptoms of exacerbation and to manage medication. We discuss these three issues in turn and suggest specific policy and practice implications of the findings. First, co-morbidity diminished patients' ability to act on risk factors, which has been noted in other literature [9,17-19]. Future management strategies and guidelines should be informed by dialogue between patients and professionals as well as lessons learnt in studies addressing specific co-morbidities clusters [19-22]. One solution to the challenge of maintaining an exercise regimen would be for cardiac and pulmonary rehabilitation programs to undergo re-design, catering to the needs of COPD and CHF participants with common co-morbid conditions such as arthritis. Policy interventions that offer incentives to rehabilitation programs could effectively initiate the required changes to increase the programs' capacity to meet more common combinations of co-morbid conditions. The success of this solution will depend on the increased understanding of co-morbidity among HCPs and their increased communication between specialities [22]. Existing initiatives such as the Enhanced Primary Care Multidisciplinary Case Conferencing and Care Plans, or The Australian Government's Health *Connect *program facilitate communication between specialities and increasing their uptake will benefit patients with co-morbid illness [23-26].

Second, co-morbidity made it difficult for patients to identify signs and symptoms of an exacerbation of an index condition. Kerr, Heisler, and Krein et al. (2007) found complications occurred when patients prioritised the self-management of one condition over another [18]. However, our findings suggest it is the complexity of the knowledge required and the confusing nature of the symptoms that prevent patients from recognising physical and psychological changes. The ability to recognise signs and symptoms of each illness is an important aspect of self-care and care planning [27,28] and patients may benefit from policy that promotes patient health knowledge through self-management planning. Much written patient information provided in primary care settings is disease-specific. Many non-government organisations are orientated towards single diseases or organs (e.g. Diabetes Australia or the National Heart Foundation). Recent policies such as the 2005 National Chronic Disease Strategy focus on common single conditions rather than co-morbid issues. All these observations reflect the dominant disease silo orientation of current Australian policy and practice [26,27].

The third limitation was that co-morbidity interfered with patient capacity to manage their medications and adhere to medication regimens. This was made worse by polypharmacy, poor medication literacy or confusion about regimens, and financial pressure [29-32]. Patients may benefit from medication education and services that address these complications [31,33]. In Australia this has been addressed through a pharmacist in-home patient medication review as part of a GP care plan called the Domiciliary Medication Management Review (DMMR). This review has had slow uptake across Australia because of pharmacist workforce shortages, pharmacists needing to be accredited before they can access the item, insufficient financial incentives, insufficient collaboration between pharmacists and GPs and insufficient promotion of the item [34]. These factors need to be addressed to support the needs of patients who have co-morbid conditions [34,35]. None of the participants in our study mentioned the DMMR, which might reflect the initiative's shortfalls.

These three difficulties in self-management, which stem from co-morbidity, have been recognised in other studies as well. The Kaiser Model of Stratified Care according to Patient Need [36] and the Wagner Model of Chronic Care concerning self-management and patient decision-making support [37], are two widely used models of care that support the promotion of patient education and collaboration between patients and HCPs for effective self-management. There is room within these models to address the needs of people with multiple conditions. Research is now needed to address specific combinations of illnesses that are known to be co-morbid and highly prevalent. This kind of research may suggest modifications to the existing chronic care models and will inform policy initiatives at national and state levels that aim to improve patients' capacities to act on risk factors, knowledge of signs and symptoms, and capacity to manage medication.

### Limitations

We did not aim for generalisability; rather, we aimed for a representative sample of patients with the three index conditions, saturation of issues raised in responses from our participants, and coherent interpretations of our data. While the research was conducted across two local sites the findings do not indicate they are site-specific.

## Conclusion

The majority of patients with DM, COPD or CHF have co-morbid conditions. At a clinical level, co-morbidity is a complication that challenges the management of chronic illness by patients and their carers. Given its frequency, future clinical policy initiatives need to move away from single illness orientation towards strategies that embrace the needs of people with co-morbid conditions to strengthen patient capacity to self-manage.

## Competing interests

The funding organisation (NHMRC) had no role in the study design, data collection, analysis and interpretation, or the writing and publication of this article. The author(s) declare that they have no competing interests.

## Authors' contributions

TJ collected, analysed and interpreted data, was heavily involved in drafting the manuscript and revising it critically for important intellectual content. Y-HJ contributed to conception and design of the study. Y-HJ collected, analysed and interpreted data, was heavily involved in drafting the manuscript and revising it critically for important intellectual content. PD interpreted data and was involved in drafting the manuscript and revising it critically for important intellectual content. NJG made substantial contributions to conception and design. NJG critically revised the manuscript for important intellectual content. MK interpreted data and was involved in drafting the manuscript and revising it critically for important intellectual content. TU contributed to conception and design. TU interpreted data and was involved in drafting the manuscript and revising it critically for important intellectual content. All authors read and approved the final manuscript.
